# Impact of dialect use on a basic component of learning to read

**DOI:** 10.3389/fpsyg.2015.00196

**Published:** 2015-03-24

**Authors:** Megan C. Brown, Daragh E. Sibley, Julie A. Washington, Timothy T. Rogers, Jan R. Edwards, Maryellen C. MacDonald, Mark S. Seidenberg

**Affiliations:** ^1^Program in Communication Sciences and Disorders, Educational Psychology and Special Education, Georgia State UniversityAtlanta, GA, USA; ^2^Psychology, University of Wisconsin-MadisonMadison, WI, USA; ^3^Communication Sciences and Disorders, University of Wisconsin-MadisonMadison, WI, USA

**Keywords:** reading, dialect, African American English, achievement gap

## Abstract

Can some black-white differences in reading achievement be traced to differences in language background? Many African American children speak a dialect that differs from the mainstream dialect emphasized in school. We examined how use of alternative dialects affects decoding, an important component of early reading and marker of reading development. Behavioral data show that use of the alternative pronunciations of words in different dialects affects reading aloud in developing readers, with larger effects for children who use more African American English (AAE). Mechanisms underlying this effect were explored with a computational model, investigating factors affecting reading acquisition. The results indicate that the achievement gap may be due in part to differences in task complexity: children whose home and school dialects differ are at greater risk for reading difficulties because tasks such as learning to decode are more complex for them.

## Introduction

In the United States, the term “achievement gap” refers to disparities in academic performance between groups of individuals. The term is mainly used with reference to racial and ethnic minority groups—African Americans, Hispanics, and Native Americans—compared to whites, but there are many other “gaps” (e.g., gender, income, first vs. later generation children of immigrants). Although the achievement gap in reading for African American children in particular has been the focus of attention from politicians, educators, and economists for many years (Jencks and Phillips, 1998; Equity and Excellence Commission, 2013), it has been a persistent, seemingly intractable problem with important consequences for individuals and society (Vanneman et al., 2009). Econometric analyses of large surveys such as the Early Childhood Longitudinal Study (ECLS; Najarian et al., 2010) suggest that multiple factors contribute to the gap, including characteristics of the child, parents, home environment, schools, and culture (Yeung and Pfeiffer, 2009). Although differences in socioeconomic status (SES) can account for much of the gap that exists in kindergarten, it does not explain the increase in the gap over the first several years of schooling (Fryer and Levitt, 2004, 2006). These types of studies also do not address the mechanisms by which such factors affect learning to read in particular.

Differences in language background may be an important contributing factor to reading acquisition and school achievement. Surveys such as the ECLS contain little information about children's linguistic background other than which languages are spoken in the home. Thus, the ECLS provides important data related to differences *between* languages (e.g., spoken at home vs. school), but does not allow examination of the impact of linguistic differences *within* a language. However, a large body of research shows that characteristics of the language to which children are exposed have enormous impact on what they learn (Hoff, 2013).

Although all humans acquire spoken language in the absence of pathology, the characteristics of people's language vary widely. Languages exhibit statistical regularities involving the frequencies and co-occurrences of sounds, words, and phrases. Children acquire knowledge of these regularities via exposure to large samples of utterances, beginning *in utero* (see Romberg and Saffran, 2010, for review). This implicit statistical knowledge is continually updated and elaborated across the lifespan through language use (MacDonald, 2013). Importantly, speech to children exhibits wide variation in quantity and quality: the sheer number of utterances, their variety and complexity, and the frequency and duration of communicative exchanges (Hart and Risley, 1995; Hansen and Joshi, 2008; Goldstein et al., 2010). Although outcomes are also affected by variation in perceptual and learning capacities, individual differences among typically-developing children are dwarfed by the impact of the much larger variation in experience (Weizman and Snow, 2001; Huttenlocher et al., 2010).

The impact of differences in spoken language skills (e.g., phonological awareness, vocabulary size, complexity of vocabulary entries) on early reading has been well-documented (McCardle et al., 2001; National Reading Panel, 2001). Less well-understood are the potential effects of another source of variability in children's language experience, the use of different dialects. Many African American children speak African American English (AAE), a major dialect of English. Dialects are variants of a language, spoken by individuals grouped by region, ethnicity, race, income, and other factors (Chambers and Trudgill, 1998). AAE is usually compared to Mainstream American English (MAE, also termed “Standard American English”; Craig and Washington, 2004), the “higher prestige” dialect (in the sociolinguistic sense) used in government, business, and education. Although discussions focus on the distinction between AAE and MAE, each dialect has regional variants as well. From a strictly linguistic perspective, AAE is unremarkable: it exemplifies very general processes by which language variation creates identifiable dialects of spoken languages. Which dialect functions as the “standard” is not a linguistic issue but rather is determined by demographic, economic, and cultural considerations.

As with the dialects that occur in other languages, AAE and MAE mostly overlap (because they are both dialects of English), but also differ with respect to elements of phonology, morphology, syntax, pragmatics, and discourse (Green, 2002). Children who use AAE in the home and community typically attend school where MAE is emphasized. Given this situation, differences between the dialects could affect a child's learning experience. As an example, consider rhyming. The ability to produce and recognize rhyming words like GOLD-BOLD is one of the foundational pre-reading skills listed in the Common Core Standards for Reading in kindergarten (Common Core Standards Initiative, 2014), a documented marker of the child's developing phonological awareness (National Reading Panel, 2001), and included in standardized assessments of early reading (e.g., Woodcock et al., 2001). However, rhyming patterns differ across dialects. For example, GOLD, BOWL, and LOW rhyme for many speakers of AAE, but not for most MAE speakers. This dialect difference illustrates two points. First, the mappings between spelling and sound differ across dialects. Whether such differences have a significant impact on children's learning is not known. Second, activities directed at developing reading readiness and beginning reading skills may function differently across dialects because of such differences. Phonological differences are particularly relevant to early reading, but other differences (e.g., in morphology and syntax) may be equally relevant to achievement outcomes.

Although strong views have been expressed by researchers from a variety of backgrounds (education, psychology, linguistics), there is little agreement as to whether differences between home and school dialects affect reading and school achievement (e.g., Goodman and Buck, 1973; McWhorter, 1997; Terry et al., 2010a; for review, see Charity et al., 2004). Historically, a major advance was achieved with the accurate characterization of the linguistic properties of AAE (Labov, 1972), refuting earlier descriptions that were inaccurate and often frankly racist (for background, see Baratz and Baratz, 1970; Smitherman, 2000; Tamura, 2002). The proper linguistic characterization of the dialect stimulated a surge of research activity and commentary (see Rickford et al., 2013, for an annotated bibliography). Much attention focused on whether differences between the dialects could have an impact on learning to read (e.g., Labov, 1966; Wolfram, 2004).

The linguistic integrity of the dialect suggested to some scholars that there would be no reason for it to function differently than other dialects in contexts such as school. For example, McWhorter (1997, p. 9) asserted that “It is a fact that Black English is not different enough from standard English to pose any significant obstacle to speaking, reading, or writing it. Black English is simply a dialect of English, just as standard English is.” McWhorter did not provide evidence supporting this assertion. However, several early studies appeared to demonstrate little effect of dialect use on reading or comprehension of spoken MAE (e.g., Baratz, 1973; Hart et al., 1980). The focus of research then shifted to other factors that might affect reading and school achievement, such as the use of culturally-relevant curricula (Ladson-Billings, 1992). Questions about the possible impact of dialect differences need to be considered further, however (Charity et al., 2004; Seidenberg, 2013; Washington et al., 2013). First, there is insufficient research on this issue using modern methods employed in other psycholinguistic studies of reading and language. The early studies purporting to show no impact of dialect on reading or school performance do not hold up well by contemporary standards. Second, there is a need to test specific hypotheses relating dialect differences to steps in learning to read. Some features of the dialect may be highly relevant to reading and others not at all. Finally, tests of any hypothesized effect of dialect need to take into account differences in dialect density, the extent to which speakers employ dialect features (Washington and Craig, 1998). The use of such features (such as rules that create alternative pronunciations or verb morphology) is optional rather than obligatory, as in other dialects. Speakers vary in the extent to which they use AAE features and the extent to which they are familiar with the mainstream dialect, and this variability should modulate any dialect-related effects. For example, Edwards et al. (2014) found that dialect density predicted children's comprehension of words spoken in MAE, and Charity et al. (2004) found that children's accuracy imitating MAE forms was a reliable predictor of their scores on standardized measures of early reading abilities.

We examined the possible relevance of dialect differences to early reading achievement by linking them to a critical step in reading acquisition, learning to decode. Decoding is the use of spelling-sound correspondences to read aloud letter strings in alphabetic writing systems. Decoding skill is strongly related to early reading achievement in English (McCardle et al., 2001; National Reading Panel, 2001) because it contributes to the development of fluent, accurate word reading, a foundation for more advanced comprehension skills. Word and non-word decoding tasks are included in standard psychometric assessments such as the Woodcock–Johnson (Woodcock et al., 2001) and CTOPP (Wagner et al., 1999). Learning to decode is a major hurdle for beginning readers because of the abstract relationship between letters and sounds (Liberman, 1973) and inconsistencies in spelling-sound mappings in English (Seidenberg and McClelland, 1989). Decoding difficulties are characteristic of younger and older struggling readers (Shankweiler et al., 1999) and developmental dyslexics (Shaywitz and Shaywitz, 2005).

We hypothesized that differences between the pronunciations of words in AAE and MAE complicate the already challenging task of learning to decode (see also Charity et al., 2004). Although many words in the two dialects have the same pronunciations at the phonemic level (e.g., CHAIR, TOWN), others differ in various ways described by Craig et al. (2003). For example, AAE allows reduction of final consonant clusters, as in TEND -/tεn/ and BEST -/bεs/, in contrast to the MAE pronunciations /tεnd/ and /bεst/, respectively. Thomas-Tate et al. (2006) found that these dialect differences had an impact on children's phonological development, with AAE-speaking children being about 1 year behind MAE-speaking children in tasks tapping implicit knowledge of final consonants. Although final consonant reduction is only one small part of AAE phonology, it has several effects that may complicate the task of learning to relate spoken and written language. First, it creates words in which a letter is not pronounced (as in TOLD-/tol/). These words deviate from the alphabetic principle that letters or combinations of letters correspond to phonemes (Rozin and Gleitman, 1977). Second, whether a letter is pronounced varies across words, creating more complex contingencies. For example, the /d/ in DOG is pronounced whereas the /d/ in TOLD can be silent. Third, for AAE speakers who are also familiar with MAE, there are many words (such as POUND) for which one spelling is associated with two pronunciations, depending on whether the final consonant is deleted or not. Finally, some final consonant deletions create homophones, as in COLD-/col/, homophonous with COAL.

These effects are not dialect specific but rather reflect general properties of English. “Silent letters” occur in words such as LISTEN and COMB. Many MAE speakers maintain alternative pronunciations of words such as OFTEN, NEITHER, and URANUS. Spoken English has hundreds of homophones, which listeners manage to comprehend quickly and accurately. The impact of the forms resulting from dialect influences is to take an orthography that already lacks transparency and consistency and make it even more so for AAE-speaking readers. The empirical question is whether these increases in opacity have a significant impact on comprehending or producing written language.

Related phenomena have been studied with other populations of beginning readers. Many studies of MAE speakers have shown that words with inconsistent spelling-sound mappings are harder to learn to decode than words with more consistent patterns. For example, spelling patterns such as -OWN have two common pronunciations (as in OWN-FLOWN-BLOWN vs. CLOWN-DOWN-FROWN). Such words produce more pronunciation errors than words with consistent spelling-sound correspondences such as -ust (Metsala et al., 1998). They also produce longer naming latencies when they are pronounced correctly, indicating interference from knowledge of the alternative pronunciation. For words that are relatively infrequent, the detrimental effects of spelling inconsistency persist into adulthood even for skilled readers (Waters et al., 1984). These studies focused on inconsistencies related to the pronunciation of vowels, but inconsistent pronunciations of the final consonant illustrated by the /d/ in BAD and TOLD in AAE could produce a similar effect.

Words that have alternative pronunciations also present difficulties for readers. For monolingual English speakers, latencies to read words such as DOVE and WIND, for which a single spelling is associated with two pronunciations, are longer than for single-pronunciation words, reflecting the added difficulty of choosing between alternative pronunciations (Seidenberg et al., 1984). Similarly, bilingual children and adults are slower to read aloud spellings that have different pronunciations in their two languages compared with single-pronunciation words (e.g., for French-English bilinguals the word LIT exists in both languages; it is pronounced /lIt/ in English but /li/ in French, meaning *bed*; Beauvillain and Grainger, 1987; Jared et al., 2012). Extending this work to bi-dialectal children, we expect that words with different pronunciations in AAE and MAE will be more difficult than ones with the same pronunciation in both dialects.

To summarize, our behavioral experiment examined the impact of dialect-related differences in pronunciation on young African American children's decoding. We also developed a computational model to investigate possible mechanisms by which these dialect differences could affect learning to decode. Similar models have been used to examine the learning of spelling-sound correspondences in MAE, the impact of inconsistent mappings, the modulating effects of frequency, and the impact of phonological deficits on learning (Seidenberg and McClelland, 1989; Harm and Seidenberg, 1999). Other, related models have been used to study the acquisition and processing of lexical knowledge in bilinguals (French and Jacquet, 2004). Modeling allows the impact of dialect differences to be investigated independent of other factors such as SES and its many sequelae, which are usually confounded with dialect use. The models also permitted us to examine how learning would proceed under different scenarios, such as use of the same or different dialects at home and in school. These “what if” simulations also provide evidence as to whether the behavioral differences we observed were due to properties of the dialects themselves or to inconsistencies created by use of different dialects. Finally, the models were used to examine how the impact of dialect differences might be mitigated.

## Experiment

The experiment investigated whether AAE use is related to children's speed and accuracy in reading single words aloud. Children read aloud Contrastive words, which are pronounced differently in AAE and MAE (e.g., POUND) and Non-contrastive words, which are pronounced with the same phonemes in both dialects (e.g., PLATE). The two sets of words were selected to be similar in other respects and thus would be expected to differ in difficulty only if a participant were familiar with the alternative pronunciations of Contrastive words.

### Methods

#### Participants

The participants were children (*N* = 22) who identified as African American, recruited from an after school program at a local community center (10 male, 12 female; ages 8.3–13.0 years old, *M* = 11.4, *SD* = 1.3). The study was conducted with approval from the Institutional Review Board at the University of Wisconsin-Madison. Informed consent was obtained from parents, and children provided verbal assent. Data from seven other children were excluded because fewer than 50% of responses were valid due to pronunciation errors or spoiled trials (e.g., voice key triggered by an extraneous sound).

#### Materials

The stimuli were the 24 pairs of Contrastive and Non-contrastive words shown in Table 1. Selection of these words was guided by knowledge of the AAE variant of the Upper Midwest of the United States from which the experiment participants were drawn. This dialect variant shares many but not all features of other AAE variants. The AAE pronunciations of the Contrastive words all exhibited a consonant cluster reduction compared to the MAE pronunciations, and they were all judged to be acceptable AAE forms by a bi-dialectal speaker from the same geographical region as the participants. This bi-dialectal speaker judged the Non-contrastive words to have the same pronunciation in AAE and MAE at the phonological level in the local dialect. Some of the Non-contrastive words had word-final consonant clusters. The *nk* cluster appeared in three words (DRANK, SINK, PINK) because this nasal-stop cluster is not reduced in AAE (Craig et al., 2003). No children produced a cluster reduction for any of these items. Similarly, three words contained a nasal-stop cluster followed by *s* (BANKS, BUMPS, TANKS). Although omission of the final -*s* is a characteristic of some AAE speakers, it is less prominent in the Upper Midwest AAE of our participants, and indeed, no child produced a reduced consonant cluster for any of these items. Two other Non-contrastive words contained a final *r* (FLOOR, AIR), which is often reduced in some variants of AAE (e.g., pronouncing FLOOR as /flo/), but this reduction is also not characteristic of the Upper Midwest AAE speakers in our sample. No children omitted the *r* in pronunciation of these words.

**Table 1:** **Stimulus words, descriptive statistics, mean naming latencies**.

**Contrastive stimuli**						**Non-contrastive stimuli**					
**Word**	**TASA**	**L**	**P**	**ELP**	**Exp**	**Word**	**TASA**	**L**	**P**	**ELP**	**Exp**
Blast	0.96	5	5	562	859	Blame	1.11	5	4	576	829
Boast	0.31	5	4	574	1125	Bumps	0.99	5	5	572	878
Bound	0.99	5	4	567	902	Brush	1.68	5	4	558	978
Build	1.98	5	4	622	972	Beach	1.92	5	3	624	926
Burst	1.43	5	4	645	971	Banks	1.26	5	5	605	894
Coast	1.57	5	4	636	1070	Crack	1.49	5	4	620	773
Drift	0.68	5	5	618	744	Drank	1.41	5	5	640	914
End	2.57	3	3	586	730	Air	2.69	3	2	588	726
Ghost	1.54	5	4	556	891	Goose	1.24	5	3	628	1002
Hind	0.91	4	4	615	757	Hush	0.80	4	3	591	737
Hound	0.87	5	4	582	872	Hatch	1.12	5	3	603	803
Lend	0.92	4	4	625	885	Lawn	1.34	4	3	583	1019
Loft	0.56	4	4	564	892	Loom	0.78	4	3	605	879
Old	3.01	3	3	563	809	Own	2.70	3	2	583	808
Pest	0.49	4	4	608	943	Peek	0.64	4	3	581	836
Pound	1.26	5	4	589	815	Plate	1.47	5	4	573	829
Sand	2.05	4	4	620	789	Sink	1.45	4	4	634	831
Spent	1.88	5	5	645	707	Stage	1.63	5	4	666	687
Toast	1.06	5	4	586	926	Tanks	1.05	5	5	554	1014
Waste	1.45	5	4	561	811	Worse	1.65	5	3	586	771
Fast	2.39	4	4	549	779	Flat	1.97	4	4	570	715
Found	2.81	5	4	622	759	Floor	2.38	5	4	603	763
Post	1.52	4	4	582	729	Pink	1.54	4	4	585	784
Dust	1.78	4	4	547	887	Drop	1.79	4	4	595	884
Mean	1.46	4.5	4.0	593	859		1.50	4.5	3.7	597	845

TASA is the log second grade frequency from Zeno (1995). L is number of letters, P is number of phonemes in the MAE pronunciation. ELP is the mean naming latency for the word from (Balota et al.'s 2007) large study with skilled adult readers. Exp is the children's mean naming latency for the item in the current experiment.

Contrastive–Non-contrastive pairs were equated on major properties that affect naming latencies: log token frequency (Zeno, 1995), number of letters, and initial phoneme. Naming latencies for the two types of stimuli taken from the English Lexicon Project database (Balota et al., 2007) did not differ, indicating that the Contrastive and Non-contrastive words were matched for naming difficulty in this large sample of adults. Table 1 also shows that pairs were matched for number of phonemes given the MAE pronunciation of the Contrastive words. AAE pronunciations were one phoneme shorter, given the final consonant reduction. Matching on the MAE phoneme lengths is thus the conservative choice, because shorter word lengths typically reduce naming latencies. We predicted that the dual-pronunciation aspect of Contrastive words would increase their difficulty over Non-contrastive words, beyond any benefit from being slightly shorter.

#### Procedure

Children were told that the experiment was about reading words out loud; dialect was not mentioned. They were seated at a comfortable distance from a computer in a quiet area. On each trial, a word appeared on the screen in lower case letters. Children were instructed to read the word aloud quickly and accurately, after which it was removed from the screen. The experimenter then pressed a key to advance to the next trial. After practice trials, participants named 48 words, with order of presentation randomized by participant. Responses were audio-recorded for later scoring of pronunciations.

Children's usage of AAE was assessed using a sentence repetition task previously developed for this purpose (Charity et al., 2004), which also correlates with children's use of AAE features in spontaneous speech (Charity, 2007). Children were instructed to repeat sentences spoken in MAE, which afforded 60 opportunities to produce a phonological or grammatical form that occurs in AAE but not MAE. Dialect use was indexed by the number of AAE forms produced. Finally, an MAE-speaking experimenter administered expressive (Expressive Vocabulary Test, EVT-2; Williams, 1997) and receptive (Peabody Picture Vocabulary Test, PPVT-4; Dunn and Dunn, 1997) vocabulary tests in order to assess general language abilities.

### Results

#### Dialect and vocabulary scores

On the sentence repetition task, children produced an average of 11 AAE features (*SD* = 4.24, range 5–20 features). The rate of AAE features here is lower than reported by Charity et al. (2004). The differences likely owe to a combination of factors. First, the children in our sample were older than the 5- to 8-year-old children in Charity et al.'s, sample; Charity et al. found that use of AAE features declined with age. Second, Charity et al. found wide variation in children's performance across their three testing locations (Cleveland, New Orleans, and Washington, DC), and so it is not surprising that a fourth location also varies. Third, not all possible AAE features in this passage identified by Charity et al. (2004) are characteristic of the Upper Midwest dialect variant, and so even heavy AAE users in our sample would not be expected to produce some AAE features.

Expressive vocabulary standard scores (EVT-2) averaged 93.55, *SD* = 10.74; receptive vocabulary standard scores (Peabody Picture Vocabulary Test, Fourth Edition: PPVT-4) averaged 94.05, *SD* = 8.50. These scores are within the normal range. AAE usage was negatively related to performance on both vocabulary tests (EVT-2: β = −0.31, *p* = 0.009; *R*^2^ = 0.29, 95% CI [−0.53, −0.08]; PPVT-4: β = −0.25, *p* = 0.001; *R*^2^ = 0.44, 95% CI [−0.38, −0.12]). Such results must be interpreted cautiously, however. These language assessment instruments evaluate use of the standard dialect. Children who knowledge AAE may be less familiar with MAE forms that are scored as correct on these vocabulary tests, and their knowledge and use of alternative words in AAE is not assessed. See Washington (2001) for discussion.

#### Word pronunciations

Children's pronunciations in the word reading task were scored by a bi-dialectal speaker. Children produced AAE pronunciations for the Contrastive words on 31.8% of the trials, and every child produced at least one AAE pronunciation for Contrastive items. Non-contrastive words were given AAE pronunciations 2.8% of the time; these consisted of vowel changes known to be used by some AAE speakers in some environments (Craig et al., 2003). Thus, aside from these few exceptions, the Non-contrastive words yielded nearly 100% MAE pronunciations, whereas the Contrastive words received AAE pronunciations about 1/3 of the time.

#### Naming accuracy

Both AAE and MAE pronunciations were scored as correct in this analysis. Responses that were not valid pronunciations of the word in either dialect, such as naming SHIFT as SHAFT, were scored as errors. Overall, 94.4% (*SD* = 6.3%) of words were pronounced correctly. We analyzed the error data with a mixed-effects logistic regression (lmer) analysis (Baayen et al., 2008; Jaeger, 2008) using the lme4 package in R (Bates et al., 2013). Random intercepts for item and subject were included, and forward selection was used to evaluate the contribution of each random slope that was included as a fixed effect and expected to influence error rates. None of the random slopes improved fit and the random effects are not presented here. As Table 2 shows, there were reliable effects of Age and EVT score (older children and children with higher EVT standard scores made fewer errors), and word frequency was also a predictor of naming accuracy (higher frequency words named more accurately). Children were also reliably more accurate on Non-contrastive words (96.6% correct, *SE* = 4.0) than on Contrastive words (91.9%, *SE* = 3.3). However, there was also a reliable interaction between contrastiveness and AAE use. The direction of this effect was that the disparity in error rates between Contrastive and Non-contrastive words grew with AAE use, such that children with higher rates of AAE features in their speech produced substantially more errors on Contrastive words than Non-contrastive words. Error rates in the two conditions were very similar for children who produced fewer AAE features.

**Table 2:** **Results of mixed-effects logistic regression model on the rate of naming errors**.

**Effect**	**Coefficient**	***SE***	***t***	***p***
(Intercept)	4.380	0.420	10.53	0.000
Contrastiveness	−0.830	0.340	−2.440	0.015^*^
AAE use	0.020	0.090	0.190	0.849
TASA log frequency	1.790	0.370	4.850	0.000^*^
Age	0.040	0.020	2.630	0.009^*^
EVT standard score	0.070	0.030	2.170	0.030^*^
PPVT standard score	0.040	0.050	0.940	0.348
Contrastiveness × AAE use	0.160	0.080	2.010	0.045^*^

* *p < *0.05**.

#### Naming times

For this analysis, all mispronounced words (*M* = 5.6%; *SD* = 6.3%) were removed. Trials on which the voice key failed to detect the response or triggered prematurely were also removed (10.2% of the data). These trials were distributed similarly across conditions (Contrastive: 10.0% of trials; Non-contrastive: 10.4%).

**Figure 1:**
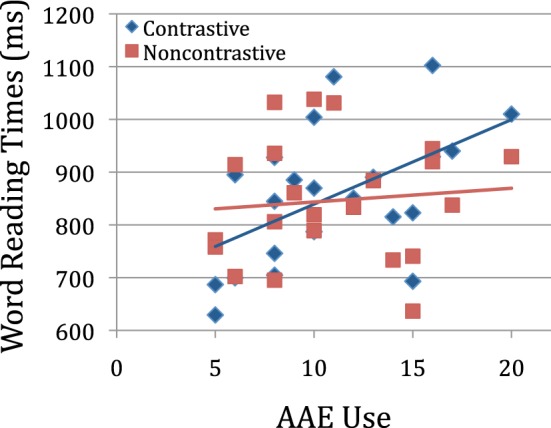
**Mean naming latencies for Contrastive and Non-contrastive words for each child, as a function of the number of AAE features children produced in the Charity et al. (2004) sentence repetition task**.

Latencies were analyzed using linear mixed models with REML estimation and included random intercepts for item and subject. Forward selection was used to evaluate the contribution of each random slope for fixed effect predictors that were expected to influence error rates. None of the random slopes improved fit and the random effects are not presented here. As can be seen from the model output in Table 3, there was a main effect of word frequency (frequent words named faster) but no main effects of participant factors (Age, EVT, PPVT, or AAE use). Mean naming latencies for Non-contrastive words (863 ms, *SD* = 273) did not differ from latencies for Contrastive words (872 ms, *SD* = 277). However, there was a reliable interaction between Contrastiveness and children's AAE usage, such that higher AAE usage predicted longer naming times for Contrastive vs. Non-contrastive words. This interaction can be seen in Figure 1, which shows Contrastive and Non-contrastive reading times for each child as a function of AAE usage. As the figure shows, the source of the interaction is increasing reading times for Contrastive words with increasing AAE usage, while reading times for Non-contrastive words do not differ with AAE usage. For children with low AAE usage, Non-contrastive words were numerically slower than Contrastive words. As this pattern reflects a small number of children, we are unsure of its interpretation, and it should be investigated further in future studies.

**Table 3:** **Summary of the coefficients from the mixed effects analysis of naming times**.

**Effect**	**Coefficient**	***SE***	***t***	***P***
(Intercept)	822.890	34.300	23.99	0.000
Contrastiveness	18.163	16.350	1.110	0.268
AAE use	2.600	8.200	0.320	0.749
TASA log frequency	−55.510	16.350	−3.400	0.001^*^
EVT standard score	3.060	3.070	1.000	0.318
PPVT standard score	−2.332	4.380	−0.532	0.595
Pronunciation	25.820	22.120	1.167	0.244
Age	−2.010	1.800	−1.140	0.255
Contrastiveness × AAE use	13.560	3.650	3.710	0.000^*^

* *p < 0.05*.

These results suggest that implicit knowledge of alternate MAE-AAE pronunciations affected children's decoding. That is, we take children's AAE use on the repetition task as an estimate of their use of AAE pronunciations in their spontaneous speech (Charity, 2007), and thus an estimate of the degree to which they have represented both AAE and MAE pronunciations of Contrastive words. Children who used more AAE features in the sentence repetition task produced more reading errors on Contrastive words compared to Non-contrastive words. Since both MAE and AAE pronunciations were scored as correct, this result indicates that the alternative pronunciations interfered with producing either of them. In addition, on correct trials, Contrastive words yielded longer latencies than Non-contrastive words for heavier AAE users, but not for the children with less AAE usage. These effects are similar to ones observed with homographs (e.g., WIND), which have two pronunciations and yield longer naming latencies than non-homographs. The results are also consistent with those found with bilinguals, arising here from differences in pronunciation of a printed form across two dialects rather than across two languages.

We next implemented a computational model to further explore the effects of dialect on reading. The model offers an opportunity to expand the notion of Contrastive words to include other words with AAE-MAE alternative pronunciations, beyond the consonant cluster reduction AAE feature that was investigated in the experiment. It also allows us to observe the effects of two pronunciations for the written code in the absence of real-world confounds such as SES, vocabulary size, and school quality.

## Simulation 1: effects of multiple pronunciations

The model was based on one developed by Harm and Seidenberg (1999) that simulated the acquisition and use of spelling-sound knowledge in MAE (see also Seidenberg and McClelland, 1989; Plaut et al., 1996). These models instantiate the theory that spelling-sound correspondences are acquired from experience, via statistical learning procedures. The models do not mimic the child's every experience; rather, they show how knowledge of the mappings between spelling and sound is represented (in a network that encodes statistical relations between spelling and sound codes), how this knowledge changes over time, and how factors such as word frequency and spelling-sound consistency affect learning and performance. The models also permit strong tests of hypotheses concerning the bases of individual differences in reading, the causes of reading impairments, and the effectiveness of different types of remediation (Harm and Seidenberg, 1999; Harm et al., 2003).

The model was a multilayer network that first learned the phonological forms of monosyllabic words (the speech phase), to approximate the child's knowledge of spoken words prior to learning to read. The model then also learned to map spellings onto phonological forms (the reading phase), while maintaining the spoken vocabulary. The model was trained on two corpora derived from MAE and AAE, for which pronunciations of about half of the words differed. Although very little data exists concerning how often and how consistently different pronunciations are used across individuals and age groups, the proportion of Contrastive words in the training corpus is probably higher than would typically be observed in the larger vocabularies of AAE-speakers. At the same time, the AAE corpus also incorporates only some of the phonological properties that differ from MAE. As such, the models cannot be taken as corresponding to any individual speaker. Rather, these corpora provide a tool for investigating how pronunciation differences based on AAE and MAE affect learning to generate phonological codes from print, and a baseline against which training involving other corpora can eventually be compared.

Three training conditions were created to address ways that dialect experience could impact learning. In the MAE Match condition, models were trained on MAE pronunciations in the speech phase and then trained to generate these same pronunciation from spellings in the reading phase; it is a “Match” condition because the same pronunciations are used in speech and reading. The MAE Match condition is designed to approximate the reading performance of a monodialectal MAE speaker. In the corresponding AAE Match condition, AAE pronunciations were used in both phases, simulating an AAE speaker who is not exposed to MAE. Unlike the MAE Match condition, this training condition is a deliberate departure from reality (insofar as AAE speakers typically are exposed to MAE). Because the two Match conditions are similar in other respects, the comparison between them provides evidence about a specific question, the difficulty of learning the two somewhat different sets of spelling-sound mappings. As noted in the introduction, the phonological reductions in AAE create more silent letters in learning to read, and the comparison between the MAE Match and AAE Match conditions addresses whether these phonological reductions and other AAE features affect learning.

Finally, in the Mismatch condition, the model was initially trained with the AAE speech corpus and then trained to map spellings onto MAE pronunciations. For about half the words in the training set, this meant learning to generate a pronunciation that differed from the AAE pronunciation learned in the speech phase. This condition is designed to approximate the situation in which a child hears and speaks AAE in the home but then is exposed to MAE in reading instruction and other classroom activities. Performance in this condition addresses the effect of exposure to multiple pronunciations and whether differences between the home and school dialects affect learning to decode.

### Methods

#### Model architecture

**Figure 2:**
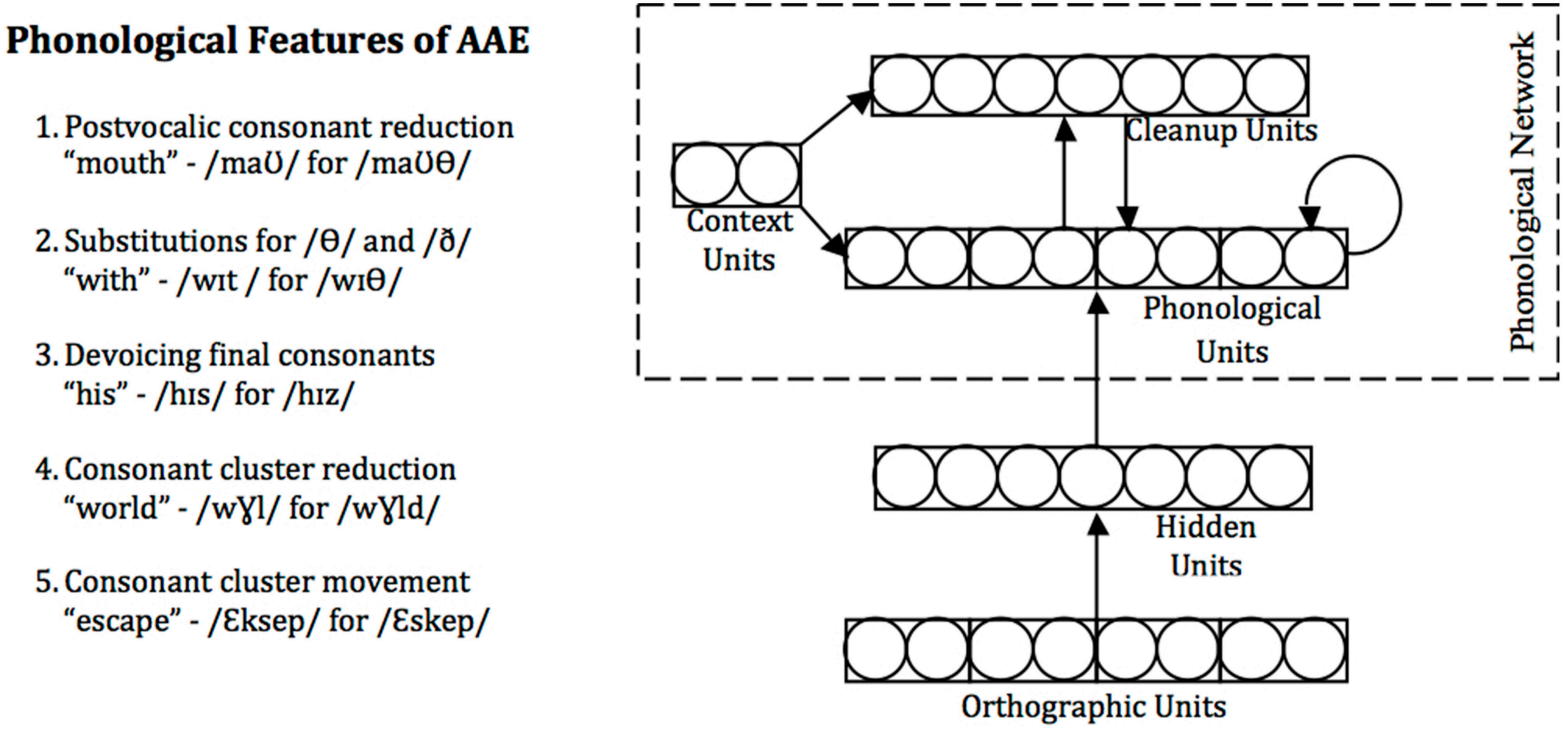
**Left:** Rules used to create the AAE corpus (Craig et al., 2003). **Right:** Architecture of the computational model. Each layer consists of a set of units. Arrows indicate weighted connections between units; all units at one layer (e.g., orthography) have connections to all units at the connected layer (e.g., hidden). Spellings are represented as patterns of activation over the orthographic units; pronunciations are represented in an analogous manner on the phonological layer. Cleanup units and interconnections between phonological units create attractor dynamics such that the model settles into a phonological code over time. Hidden units allow the model to represent the complex contingencies between orthography and phonology that exist in English. Functions of the context units are discussed in connection with Simulation 2.

The model consisted of a phonological network representing pronunciations and additional units used in the reading task (Figure 2). Pronunciations of words were represented on the phonological layer using localist representations of phonemes in 10 positional slots, with centering on the vowel. These units were connected to one another and to a second “cleanup” layer, forming an attractor network in which the steady states correspond to learned phonological forms (see Plaut et al., 1996). The context units shown in Figure 2 were used only in Simulation 2, described below.

The orthographic layer represented words in a similar fashion, using localist representations of letters in each of 12 positional slots, again centered on the vowel. The orthographic layer was connected to the phonological network via a set of hidden units. The numbers of units per layer was as follows: Orthography, 312; Hidden, 50; Phonology, 390; Cleanup, 20; Context (for Simulation 2), 2. See Appendix for further details.

#### Training

The MAE Match, AAE Match, and Mismatch conditions were created by using two training corpora reflecting differences between MAE and AAE pronunciations. The MAE corpus consisted of the 1709 monosyllabic words, excluding proper names that had frequencies above 10 in second grade norms (Zeno, 1995). School-age children know more words than this corpus, but the set included most common vocabulary words and incorporated a range of patterns that reflect the phonological knowledge of MAE-speaking children. MAE pronunciations were taken from the CMU Pronunciation Dictionary (http://www.speech.cs.cmu.edu/cgi-bin/cmudict).

The AAE corpus contained the same 1709 words. It was created by changing the MAE pronunciations using five common AAE rules shown in Figure 2 (Craig et al., 2003), and described in more detail in the Appendix, which affected 866 words (51%). When multiple rules could apply to a word, one rule was chosen at random. The AAE corpus was constructed with the goal of instantiating a variety of differences between the dialects in order to assess their impact. It incorporated a wider range of phonological differences between MAE and AAE pronunciations than did the Contrastive stimuli in the naming experiment, which focused solely on word-final consonant cluster reduction. Moreover, because only one of the AAE rules in Figure 2 was applied to each eligible word, there was variability in the appearance of AAE features in the AAE corpus. For example, for the word *third*, both rule 2 (substitutions for the /θ / in the word onset) and rule 4 (consonant cluster reduction) could apply, and one was chosen at random. Thus, the model was exposed to some variability in AAE feature use.

As with the MAE corpus, the AAE corpus reflects key elements of an AAE-speaking child's phonological knowledge, but does not fully represent AAE phonology or the knowledge of individual speakers. Thus, both corpora should be taken as approximations that capture some important phonological characteristics of the dialect.

Each condition (MAE Match, AAE Match, and Mismatch) was run three times using different sets of random initial weights on connections with words from the training sets presented in different random orders. Training consisted of two phases. In the speech phase, phonological codes for words were activated on the input phonological layer and the models were trained to maintain these patterns after the inputs were removed. After accuracy reached 95% in the speech phase, reading trials were introduced in the second phase, interleaved with additional speech trials. This procedure is broadly consistent with the interleaving of speech and reading activities in children's experience, and obviates the “catastrophic interference” effect (McCloskey and Cohen, 1989) that occurs when experiences are strictly blocked (Hetherington and Seidenberg, 1989). In the MAE Match and AAE Match conditions, the models learned pronunciations from one dialect in the speech phase and were then trained to generate these same pronunciations from spellings in the reading phase. In the Mismatch condition, models trained with the AAE corpus learned to map spellings onto MAE pronunciations.

In both phases, the training procedure involved a series of trials. The model was presented with an input pattern (e.g., the spelling of a word) and generated phonological output as determined by the current values of the weights. Learning involved adjustments to the weights based on the discrepancy between the computed output and the correct, target pattern, using the backpropagation algorithm (Rumelhart et al., 1995). This error-driven learning procedure allows the model to gradually find a set of weights that supports accurate performance on the training patterns; see Harm and Seidenberg (1999) for discussion of how this procedure relates to children's learning. The learning algorithm uses a uniform procedure in which the correct pattern is provided on every trial, clearly a simplification compared to children's more variable experience. However, this procedure also makes learning more difficult than for children because it leaves out other types of knowledge and experiences. For example, the model does not include a representation of semantics or incorporate episodic encoding of experiences, one of two complementary learning systems in the brain (McClelland et al., 1995). Research on computational learning algorithms also suggests providing veridical feedback on every trial may be less efficient than procedures that provide more varied types of feedback (Gibson et al., 2013). Thus, the training procedure is useful for exploring the complexity of a learning problem, general developmental trends, and variables that create differences in difficulty across different types of items or groups of individuals, but it does not closely simulate the experience of individual learners.

In the speech phase, each word was presented once per epoch. Weights were adjusted on each trial, with the cross-entropy error scaled by the square root of that word's 2nd grade frequency (Zeno, 1995) to approximate differences in frequency of exposure to words. For reading trials, error was scaled as 1/10 of the frequency used for speech, to approximate the fact that beginning readers hear words much more often than they read them. Models were run for 1000 epochs on the reading phase. Additional details concerning the training procedure are given in the Appendix.

### Results and discussion

**Figure 3:**
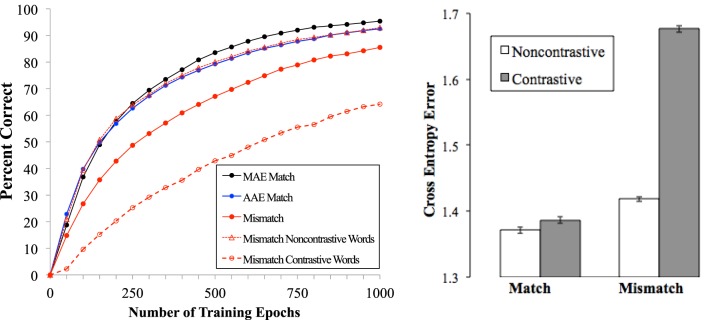
**Left graph:** Performance of the model over the course of training in the three training conditions as well as performance for Contrastive and Non-contrastive words in the Mismatch condition. **Right graph:** Cross-entropy Error for Contrastive and Non-contrastive words in the MAE Match and Mismatch conditions after 1000 epochs of training.

The primary data concern performance on reading trials as a function of type of speech training. Results averaged across the three runs in each condition are reported. In assessing the model's reading performance, the phonological output activated by a spelling pattern was scored as correct when the units corresponding to all phonemes were activated above 0.75 and units for all other phonemes were below 0.25. The left panel of Figure 3 shows this accuracy measure as a function of amount of training in the reading task and type of input in the speech phase.

In analyzing the model's performance, our first question was whether the phonological reductions and other aspects of AAE phonology by themselves increased difficulty in the reading task. A comparison of the MAE Match condition in Figure 3 (black line) and AAE Match condition (blue line) suggests that the answer to this question is, very little. In the MAE Match condition, the model reached 95.4% accuracy on the reading task with 1000 epochs of training. Performance in the AAE Match condition was similar, but learning was somewhat slower and accuracy was 92.5% at 1000 epochs. This slightly worse performance was mainly due to words with final consonant deletions, which create more silent letters in AAE corpus. The AAE Match model must learn to inhibit activation of the final phoneme for these words, which requires additional training. This difficulty is compounded by the fact that final consonant deletion is contingent on other properties of a word. For example, in TEST, the first /t/ cannot be deleted, whereas the second one can. The AAE-Match model therefore learned more slowly, but it had nearly caught up with the MAE-Match condition by the end of training. These results suggest that the phonological properties of AAE incorporated in the training set had only a minor effect on learning spelling-sound mappings.

Our second question concerned the effects of exposure to multiple pronunciations, instantiated in the Mismatch condition. As seen in the left panel of Figure 3, the model learned much more slowly in the Mismatch condition (solid red line) than in either of the Match conditions, taking 1000 epochs to achieve the accuracy level that the Match models reached with about half as much training. These results suggest that the mismatch between the two dialects affects learning spelling-sound mappings much more than the properties of either dialect. The decrement in the Mismatch condition was due to the Contrastive words in the training set. The dashed lines in the left panel of Figure 3 show that in the Mismatch condition, performance on the Non-contrastive words was nearly identical to that in Match conditions, but performance was much poorer on Contrastive words. Having learned the AAE pronunciation /bεs/ (BEST) and /dIs/ (THIS) in the speech phase, the Mismatch model had to learn to produce the MAE forms /bεst/ and /ðIs/ for these words on reading trials. This additional learning was not required in the Match conditions. As seen in the figure, the penalty for Contrastive words was substantial: accuracy after 1000 epochs of training was comparable to accuracy on Non-contrastive words after only 250 epochs.

By the end of the reading phase, the MAE Match, AAE Match, and Mismatch models generated accurate pronunciations for most words (95.4, 92.5, and 85.5%, respectively). However, even when pronunciations were correct there was variability in how closely the computed phonological output matched the correct pattern, indexed by the magnitude of the cross-entropy error. Error magnitude in a model is related to average naming latencies in adult readers (Plaut et al., 1996; Harm and Seidenberg, 1999), with harder words producing larger model error scores. To examine the effect of multiple pronunciations using the cross-entropy error measure, 378 pairs of Contrastive and Non-contrastive words were selected from the training corpora. Reflecting the methods in the experiment above, these pairs were equated on the same lexical variables as the experiment stimuli; the key differences in the model test were that many more pairs (378) were used to test the model than with children, and the Contrastive words for the model included pronunciations generated by all five rules in Figure 2, while the Contrastive words in the experiment were all items permitting consonant cluster reduction.

Mean cross-entropy error in the reading task was computed for all 378 word pairs. Cross-entropy error was lower overall in the MAE Match condition than in the Mismatch condition (Figure 3, right). Contrastive words had much higher error than Non-contrastive words in the Mismatch model, with little difference between the two word types in the MAE Match model (with similar results for the AAE Match condition). These results are consistent with the behavioral data presented above. The Contrastive and Non-contrastive stimulus words in the experiment were equated on many other factors known to affect reading difficulty and were chosen to be equally difficult for speakers of MAE. Thus, the factor that makes the Contrastive words more difficult, for both the model and for higher AAE-use children in the behavioral experiment, is having different pronunciations across the dialects.

Beyond its relevance in showing that it is pronunciation mismatch, not AAE itself that is the main source of difficulty for the model, the AAE Match condition is also relevant to an educational issue, whether teachers should correct children's AAE pronunciations in the classroom (Labov, 1966; Goodman and Buck, 1973). The fact that performance in the Mismatch condition was much poorer than in the AAE Match condition (as well as the MAE Match condition) is consistent with the conjecture that learning is easier if AAE speaking children are permitted to use AAE pronunciations, rather than having MAE pronunciations provided as corrections when they are reading aloud. Repeated correction of a child's use of AAE phonology might also be contraindicated by its potential negative impact on children's attitudes about their language and on motivation to learn (Seymour and Seymour, 1977; Ladson-Billings, 1992). Our models do not address these socio-cultural issues, and indeed the AAE Match condition instantiated in the model is an idealization in which only AAE pronunciations are utilized—in effect simulating a situation in which the MAE dialect does not exist anywhere in the child's experience. The reality for AAE-speaking children is different, involving varying amounts of exposure to and knowledge of MAE. Thus, even if AAE pronunciations are not corrected in the classroom, children may gain such feedback from other experiences, such as hearing MAE pronunciations and recognizing mismatches with their own speech. Social and cultural expectations about the use of mainstream vs. minority dialects in school are also relevant to classroom practices^1^.

In summary, Simulation 1 created a clear test of some effects of dialect on learning to read, setting aside many factors that are confounded with dialect use in naturalistic settings. The results suggested that AAE phonology by itself, although it makes the spelling-sound correspondences more inconsistent than the (already inconsistent) correspondences in MAE, increases the difficulty of learning these mappings by only a small amount. The existence of two pronunciations for Contrastive words, however, yielded a substantial burden for both the model in the Mismatch condition and the bi-dialectal children in the experiment. Results of this sort are familiar from studies of bilinguals, for whom “Contrastive” words are pronounced differently in two languages.

Two main mechanisms appear to underlie these effects. First, the number of unique phonological word forms to learn is larger in the Mismatch condition (all of the alternative pronunciations of the Contrastive words). Second, the model had to learn and maintain distinct representations of overlapping forms such as /bεs/ and /bεst/. This was particularly difficult because the model had learned the AAE pronunciations to high degree of accuracy in the speech phase, and because the model did not have a basis for treating /bεs/ and /bεst/ as related (i.e., as different pronunciations of the same word). In short, the Mismatch Model performed a more complex learning task than the Match models.

Simulation 2 examined two additional factors. First, many children are exposed to both dialects prior to school, in varying proportions. Children who are already familiar with the alternative pronunciations of words may have less difficulty learning to use MAE in learning to read (and other classroom activities). We created a Bi-dialectal condition, in which the model was trained to produce both AAE and MAE pronunciations during the speech phase, to examine this possibility. Second, children learn words in contexts that convey information about the existence of dialects, the differences between them, and the conditions under which they are used. Many speakers successfully learn to represent both dialects and switch between them (Terry et al., 2010b), although the conditions that promote learning both codes and individual differences that affect outcomes are not well-understood. The Simulation 1 results show that learning the alternative pronunciations and their relations to spelling is a more complex task, requiring additional learning trials. In Simulation 2, we examined whether the impact of the increased complexity of the task is mitigated by introducing the alternative pronunciations earlier (before the reading phase) and providing contextual cues for using AAE or MAE.

## Simulation 2: Bi-dialectal experience and contextual cues

The Bi-dialectal models in Simulation 2 used the same architecture as in the first simulation and again had a speech training phase followed by a reading phase with continued speech trials interleaved with reading. The training of the Bi-dialectal models was changed so that the models produced both AAE and MAE pronunciations in the speech phase, followed by learning to map spellings onto MAE pronunciations in the reading phase. MAE pronunciations were used in the reading phase both because schools emphasize using MAE, and to permit comparisons to the results of the Mismatch condition in the previous simulation, which showed that learning to produce MAE pronunciations on reading trials after AAE exposure in the speech phase was difficult. The Bi-dialectal conditions thus addressed whether production of MAE pronunciations prior to the onset of reading would be helpful in making the transition to MAE usage in reading.

### Methods

All models in this simulation received AAE input during the speech phase, using the AAE training set as in Simulation 1. For Non-contrastive words in the training set, the model was presented with input and had to maintain the pronunciation, as in the previous simulations. For Contrastive words, the model was presented with the AAE pronunciation and had to either maintain the AAE pronunciation or produce the MAE pronunciation. Each pronunciation target (AAE or MAE) was assigned half the overall frequency of the word, meaning that for Contrastive words, the model had to produce AAE pronunciations approximately half of the time and MAE pronunciations the other half. This procedure created variable pronunciations for the Contrastive words and gave the model experience producing the MAE pronunciations before the onset of reading.

Three Bi-dialectal conditions were developed, differing only in the use of the context units shown in Figure 2, which provided contextual cues to help the model distinguish AAE and MAE pronunciations. These two units indicated whether the model should produce AAE or MAE and served as proxies for a variety of cues that allow speakers to learn alternative dialects and switch between them. The context units were not used in the speech phase for any of the models, and thus all models had identical pre-reading experience in this simulation. In the Early Context condition, the context units were used at the onset of the reading phase. For all reading trials, the MAE context unit was on, indicating that the model should produce an MAE pronunciation for the print input. The MAE unit was also turned on during the interleaved speech trials for Non-contrastive words (which are pronounced the same in both MAE and AAE). The MAE context unit was also on for Contrastive words that were supposed to be given MAE pronunciations in speech trials, and the AAE unit was on for Contrastive words for which the Model was supposed to give an AAE pronunciation. The context units had the same effect in the Late Context condition, except that they were not used until halfway through the reading phase. Finally, in the No Context condition, the context units were never used for any reading trials or speech trials in the reading phase.

The number of epochs in the speech training phase was matched to the number of epochs used in Simulation 1. Unlike in the previous simulation, the Bi-dialectal models did not reach the 95% speech reproduction accuracy criterion with this amount of speech training because the model had more phonological word forms to learn (because it had to produce both MAE and AAE pronunciations of Contrastive words), in the same amount of training epochs. The Bi-dialectal models were then trained to produce MAE pronunciations in the reading phase for 1000 epochs, as in Simulation 1.

### Results and discussion

**Figure 4:**
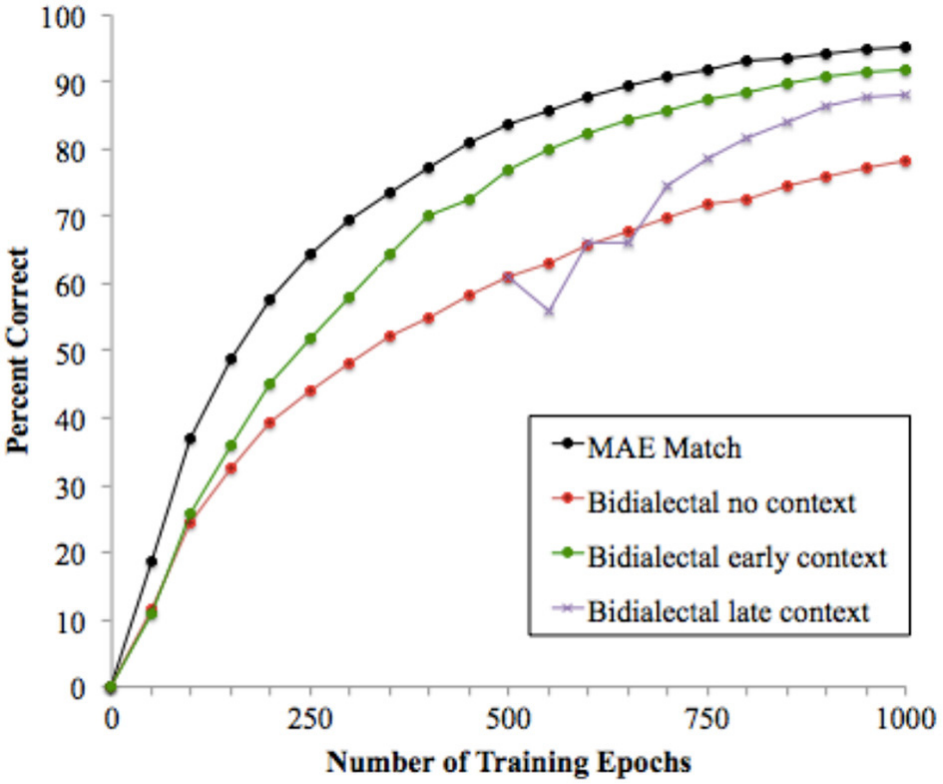
**Model performance on the reading task in the Bidalectal conditions compared to the MAE Match condition from Simulation 1**.

Reading performance was scored as in the previous simulation. Figure 4 shows percent correct performance in generating MAE pronunciations on reading trials, with the MAE Match condition from Simulation 1 included for comparison. The figure shows that on the reading task, learning was slower in all of the Bi-dialectal conditions than in the MAE Match condition. The Bi-dialectal conditions were harder for several reasons. In the speech phase, the model had a larger number of distinct phonological patterns to learn than in Simulation 1, but with the same number of training trials, which resulted in poorer speech performance. Learning to represent overlapping pairs such as /bεs/ and /bεst/ was particularly difficult; the attractor dynamics in the phonological network created a tendency to complete the pattern /bεs/ based on what was learned from training on /bεst/ and other words with the final /t/. The reading trials were also more difficult: the model had to both consolidate the MAE pronunciation and learn to generate it from the word's spelling, while training on both MAE and AAE pronunciations continued during the interleaved speech trials. The net result was that learning also occurred more slowly on the reading trials.

These effects were greatly ameliorated by providing context cues. In the Early and Late Context conditions, the contextual cue was provided for interleaved speech trials beginning at epoch 0 in Figure 4 for the Early Context Condition and at epoch 500 for the Late Context condition. Although the prior speech phase was identical for these models and the reading trials were identical (with the context units always signaling an MAE pronunciation), context cues during the speech trials varied within the reading phase, indicating either AAE or MAE pronunciations for Contrastive words. Figure 4 shows that including the context cue was helpful, with both Context conditions showing better reading performance than the No Context condition. The Early Context condition yielded reading performance very close to the MAE Match condition at the completion of the 1000 epochs of reading training; the Late Context cues were also effective, although additional training trials would be needed for performance to fully catch up.

These results suggest that cues to the alternative pronunciations in speech has an impact on learning to decode. Merely introducing both pronunciations early in training (as in the speech phase in these models) did not improve reading performance in the simulations. However, the two Context conditions show that additional information that helps the model partition the two dialects greatly improves reading performance, particularly in the Early Context condition. Again it should be noted that the models are simplified in many respects. All of the information relevant to learning about dialects, their properties, and the conditions under which they are used was captured by a single context cue. Moreover, the cue was wholly reliable and unambiguous, whereas the cues that exist in naturalistic contexts are not. The contextual cues were also introduced relatively late, at the onset of reading, whereas many children will have begun acquiring conscious or unconscious knowledge about dialectal variation earlier. Nonetheless, the main results are clear: Considered just in terms of the complexity of what has to be learned, the existence of alternative pronunciations complicates both the speech and reading tasks, and the provision of dialect-distinguishing cues is helpful.

## General discussion

We conducted one experiment with young readers and two computational simulations investigating the role of dialect on learning to read English. Both the behavioral and modeling evidence indicate that knowledge of alternative dialects affects acquisition and use of spelling-sound knowledge, an important component of reading. The experiment and simulations show that the locus of these effects is the Contrastive words, which have different presentations in the two dialects, whereas Non-contrastive words are largely unaffected by knowledge of two dialects. Previous research has shown that spellings that are associated with different pronunciations are harder to pronounce. In English, the effect occurs with homographs such as WIND and DOVE (Seidenberg et al., 1984); in bilinguals it occurs with words that have different pronunciations in different languages (e.g., COIN in French and English; Beauvillain and Grainger, 1987; Jared et al., 2012). In these cases, two semantically unrelated words with different pronunciations happen to be spelled the same. Contrastive words in AAE have a single meaning, but as in the other cases, a single spelling is associated with two pronunciations, increasing the difficulty of reading aloud. Thus, the effects of dialect can be understood as the natural consequence of the added ambiguity of the mapping between spelling and pronunciation for a subset of words.

The computational simulations produced similar effects, in models that excluded factors such as SES, school or home environment, and intelligence. The computational results suggest that the existence of two pronunciations for a word creates additional complexity both with respect to spoken language (learning different pronunciations of a contrastive word in the Bi-dialectal models in Simulation 2) and in learning the relations between spelling and phonology. Other factors that may affect performance need to be considered in future behavioral and computational work. Among the most important are (a) other phonological differences between dialects; (b) individual differences in dialect density, the extent to which an individual uses AAE features; (c) impact of vocabulary size and quality; and (d) the role of semantics in linking different pronunciations. It would also be important to address contextual cues in a richer way, taking into account their real-world variability and developmental changes in children's capacities to utilize such information. The models demonstrate that the knowledge of variable pronunciations can affect learning, but they do not predict outcomes for individuals.

The fact that the model, which excludes many factors that affect children's school performance, nonetheless reproduces the difference between Contrastive and Non-contrastive words permits some specific, though tentative inferences about the impact of dialect on early reading. The effect was not due to properties of AAE. The AAE Match model in Simulation 1 performed nearly as well as the MAE Match model, indicating that the additional irregularity from consonant deletion and other aspects of AAE phonology had a minimal effect on reading performance. The effect was not due to characteristics of children such as IQ, or to environmental factors such as differences in instruction or opportunity, none of which were incorporated in the model. Rather, the effects emerge from conflicts between the dialects which are relevant because of social and cultural conditions governing their use, specifically the fact that MAE is the dialect of instruction Children who mainly speak AAE and then are expected to use MAE in reading and other school activities have more to learn than children who only use the mainstream dialect. Results from the Mismatch condition suggest that the additional load is substantial.

Our results relate to several issues that have contributed to controversies about dialect differences and the achievement gap. Previous research of the impact of dialect differences on reading yielded mixed results. Many studies have compared the overall reading performance of children who spoke either AAE or MAE (e.g., scores on tests of passage comprehension) rather than testing hypotheses about the impact of dialect properties. There are multiple differences between MAE and AAE, only some of which may be relevant to reading and school performance. We focused on a specific characteristic linked to a specific component of learning to read, decoding. Our hypotheses about the possible relationship between dialect and decoding were motivated by extensive research on properties of spelling-sound mappings in English that affect decoding in children and adults. This research only addressed a single dialect difference. Other theory-driven hypotheses relating dialect properties to reading and school achievement should be addressed in future research.

The present results are also relevant to questions about the impact of dialect on reading compared to differences in knowledge of spoken language that are not dialect related. Reading acquisition is strongly related to knowledge of spoken language, including vocabulary and phonological awareness (National Reading Panel, 2001). AAE speakers' poor reading achievement could reflect weaknesses in these areas, as in MAE speakers, rather than dialect *per se* (Terry and Scarborough, 2011). Weaker spoken language skill is also associated with lower SES, which includes many AAE speakers.

Dialect use and spoken language skills are not mutually exclusive (Terry and Scarborough, 2011; Seidenberg, 2013); both are likely to contribute to reading outcomes (Edwards et al., 2014). The decoding effects that we have observed clearly derive from dialect differences, however: First, in both the children and in the simulations, the effects are limited to Contrastive words for which the ambiguity of spelling-sound mapping increases difficulty. Second, the effects of dialect use in the children arise even after vocabulary size is taken into account in the analyses. Third, in Simulation 1, the effects arise in the dialect Mismatch model despite equating the “richness” of the input—the AAE Match, MAE Match, and Mismatch conditions were given equivalent “experience,” yet only the Mismatch condition performed poorly, and only for Contrastive words. These results suggest that the effects of knowing multiple dialects are substantial, and it will be important to determine the degree to which individual difference and environmental factors not considered here exacerbate or mitigate these effects (Washington et al., 2013).

Finally, our results can be related to other recent studies attempting to identify factors contributing to the achievement gap. Econometric analyses suggest that the reading gap in kindergarten children can be explained by factors related to SES. However, they do not explain why the gap grows larger over the first few years of schooling (Fryer and Levitt, 2006). Importantly, the large datasets on which such analyses are based do not include measures of characteristics of the child's speech and the spoken language environment, including the use of a non-mainstream dialect. The gap's increase may be due in part to the mounting impact of linguistic factors as curricular demands increase.

In summary, our research pinpoints how a difference between dialects can affect acquiring an important reading skill. Whereas, much of the language-based debate concerning the achievement gap has addressed whether to characterize weak oral language skills as “deficits” (see Hoff, 2013, for review), our studies investigate how variability across dialects affects the complexity of learning to read. The results suggest that the AAE learner's task is literally more difficult than for an MAE speaker. Children are nonetheless evaluated against the same achievement milestones. Given these differences in task complexity, an “achievement gap” often ensues, placing children at risk for educational failure. Although we deliberately set aside SES, oral language skill, and other factors in order to focus on the effects of dialect, it is likely that the dialect-related differences observed here are exacerbated in children with weak oral language skills and other challenges.

### Conflict of interest statement

The authors declare that the research was conducted in the absence of any commercial or financial relationships that could be construed as a potential conflict of interest.

## Footnotes

^1^These effects can be understood by considering an analogous phenomenon, speakers' attempts to learn phonological contrasts that do not occur in their native language. A classic example is the /r-l/ distinction that occurs in English but Japanese. Through intensive, structured training, adult Japanese speakers can greatly improve their abilities to hear and produce the /r/ and /l/ phonemes (McCandliss et al., 2002). However, the benefits of training are difficult to maintain because speakers use Japanese outside the laboratory context, reinforcing the native phonological system. The interleaving of experiences with the two phonological systems works against acquiring the new phonological contrast. Similarly, children may benefit from the non-correction of AAE pronunciations in the classroom, but the impact may be vitiated by continued exposure to and use of MAE pronunciations in other contexts.

## Appendix

### Computational models

#### Architecture

Number of units per layer were: Orthographic (312), Hidden (50), Phonological (390), Cleanup (20), Context (2). There was complete feed-forward connectivity between nodes in adjacent layers; units in the phonological layer were also connected to each other. The effect of this pattern of connectivity is to create a feed-forward network with a phonological attractor that settled into a target pattern over time. Connection weights between layers were initialized by randomly sampling values from a uniform distribution in the range [−0.5, 0.5]. Connection weights between phonological output nodes were randomly initialized within the reduced range of [−0.1, 0.1]. Weights on connections from a node back to itself in the phonological layer were set to 1 and not trained. This prevented an output node from keeping itself active during phonological learning (see Harm and Seidenberg, 1999, for details).

#### Input and output representations

Orthographic and phonological representations for words were created using methods derived from Harm and Seidenberg (1999). There were 312 units in the orthographic layer (12 letter positions × 26 possible letters). These codes were vowel-centered, so that the fourth slot was filled with the left-most vowel of a word. A word's phonology was represented similarly, with nodes coding phonemes rather than letters (10 phoneme positions × 39 possible phonemes = 390 units).

#### Processing dynamics

A trial began when the activation of an input pattern was hard clamped to the appropriate layer (phonological on speech trials, orthographic on reading trials) for four ticks of time. Activation was propagated forward over a sequence of 12 ticks of time. The change in a unit's net input from one tick to the next was

(A1) $$\Delta I_{j}^{\left[t\right]}=\Delta t\left(\sum w_{ij}\;a_{i}^{\left[t\right]}-I_{j}^{\left[t-1\right]}\right),$$

where Δ*t* was an integration constant fixed at 0.5, *w_ij_* was the connection weight from unit *i* to unit *j, a_i_^[t]^* was the activation of unit *i* on the current tick, and *I_j_^[t−1]^* was the net input to unit *j* on the previous tick (net inputs were initialized to zero on the first tick).

Output activation, *a_j_*, of units inside the phonological network was given by

(A2) $$a_{j\;}=1/1\left(1+e^{-I_{j}}\right),$$

The logistic function was used because presence or absence of a phoneme was locally coded as a 1 or 0, respectively. Elsewhere in the network, output activation was given by

(A3) $$a_{j}=\tanh(I_{j}),$$

as in Harm and Seidenberg (1999).

Error was computed as a frequency scaled cross-entropy error function (Rumelhart et al., 1995) with a linear output cost,

(A4) $$\begin{array}{l} Error=-\sum_{t}frequency\sum_{i\;\in \;Phon}T_{i}\log(O_{i})\\ \;+\;(1-T_{i})\log(1-O_{i})-O_{i}, \end{array}$$

where *O* is the output of a phonological node *i*, and *T* is its Target state. The error for each speech trial was scaled by the square root of that word's 2nd grade frequency (*1*) to approximate differences in exposure to words. For reading trials, error was scaled as 1/10 of the square root of the frequency, in order to capture the fact that children hear more words than they read. The output cost (the final *O_i_* in Equation A4) biased weights to deactivate nodes. This deactivation bias created pressure for learning to find weights that would allow phonological representations to be maintained (*2*).

In each training epoch, all training patterns appeared once in random order. Error across the phonology output units was tabulated in the last few ticks of the settling process (ticks 9–12), then back-propagated to the hidden units at the final tick as follows:

(A5) $$\delta_{j}={f^{\prime }}_{j}(net_{j})\sum_{i\;\in \;P_{j}}\delta_{i}w_{ij}$$

so that the error on each node *j* was a function of the error on its posterior nodes, the weights connecting these nodes, and the derivative of the node's activation function. After accumulating error signals across all the items in the training corpus, connection weights were changed according to the backpropagation algorithm,

(A6) $$\Delta w_{ij}=\eta \sum \partial Error/\partial w_{ij}+\chi \Delta w_{ij}^{\mathrm{Pr}ev},$$

where η was a learning rate set to 0.00001 during phonological learning and then increased to 0.0001 during reading acquisition. The momentum parameter χ was set to 0.8, which made each weight's change a partial function of its change on the previous training epoch.

#### Training corpora

The MAE corpus was developed as described in the main text. AAE pronunciations were created by applying the five AAE rules shown in Figure 2 to the MAE pronunciations. Each Contrastive word (i.e., words for which one or more of these phonological rules could apply) received only one transformation. When multiple features could be applied to a single word, one feature was applied at random. The rules were instantiated as follows:

1. Postvocalic consonant reduction: Final consonants, following a vowel were deleted.
2. Substitutions for /θ/ and /ð/: Initial phonemes /θ/ or /ð/ replaced with /d/ phoneme. Final phonemes /θ/ or /ð/ replaced with /t/.
3. Devoicing final consonants: Final phonemes /b/, /d/, /g/, /v/, or /z/ replaced with /p/, /t/, /k/, /f/, and /s/, respectively.
4. Consonant cluster reduction: Final phonemes /t/ and /d/ deleted from consonant clusters.
5. Consonant cluster movement: The phoneme sequence /sk/ was replaced with /ks/.

Non-contrastive words, ones for which none of the above rules applied, were presented with the same pronunciation as in the MAE corpus.

#### Training procedure

On speech trials, each word was processed for 12 time ticks, with activation propagating between connected nodes and layers on each tick. Phonological input representations were provided to the model for the first four ticks and phonological target representations were applied on the final four ticks, which created pressure to maintain the phonological input pattern over time. The difference between actual and target outputs provided an error signal used to alter connection weights within the phonological network. The input and target patterns were the same in the single-dialect (MAE or AAE) models. In the bi-dialectal models in Simulation 2, the input was AAE, and the target output could be either AAE or MAE.

During reading trials, an orthographic input representation was provided for the first four ticks and the target phonological representation on the final four ticks. Error was calculated as on the speech trials. For the MAE Match, AAE Match, and Mismatch models in Simulation 1, the first phase ended when performance on speech trials reached 95% correct. The bi-dialectal models in Simulation 2 learned phonology more slowly; in these cases, the second phase began after the same number of training epochs as a paired MAE Match model.

#### Testing procedures

The results reported for every modeling condition are the average performance of three independently trained models. As in training, orthographic or phonological inputs were provided to each model for four ticks. Activation continued to propagate through the network until the 12th tick, when the activation on the phonological layer was assessed as the model's output. Output was scored as correct only when the nodes corresponding to all phonemes in the word, and no other nodes, were activated. A node was considered activated if its output was greater than 0.75 and deactivated if less than 0.25, intermediate values were considered unsettled and incorrect.

The cross-entropy error in the output pattern for a word is a continuous measure of that item's processing difficulty, correlated with behavioral naming latencies (Plaut et al., 1996; Harm and Seidenberg, 1999). Cross entropy error was computed on the final tick.

(A7) $$Error=-\sum_{i\;\in \;Phon}T_{i}\log\left(O_{i}\right)+\left(1-T_{i}\right)\log\left(1-O_{i}\right)$$

Error was calculated after 1000 epochs of training for all models in Simulation 1, when their average accuracy was similar to that of low and high AAE users in the behavioral experiment. Average cross entropy error was also computed for a subset of 378 pairs of contrastive and non-contrastive words that were equated on the same variables used to equate stimuli in the behavioral study and which are known to affect naming latency: log token frequency (Carroll et al., 1971), number of letters, and initial phoneme. The cross entropy error analyses included only output coded as correct on the above criterion. A pair of items (*M* = 17.6%) was excluded from cross entropy analyses if the computed output did not match either the AAE or MAE pronunciation.
